# Medical gas plasma technology: Roadmap on cancer treatment and immunotherapy

**DOI:** 10.1016/j.redox.2023.102798

**Published:** 2023-06-27

**Authors:** Sander Bekeschus

**Affiliations:** a ZIK *plasmatis*, Leibniz Institute for Plasma Science and Technology (INP), Felix-Hausdorff-Str. 2, 17489, Greifswald, Germany; b Clinic and Policlinic for Dermatology and Venerology, Rostock University Medical Center, Strempelstr. 13, 18057, Rostock, Germany

**Keywords:** CAP, Cold atmospheric pressure plasma, Cold physical plasma, Non-thermal plasma, Oncology, Plasma medicine, Reactive oxygen species, Redox medicine, Tumor immunology, Tumor-infiltrating leukocytes

## Abstract

Despite continuous therapeutic progress, cancer remains an often fatal disease. In the early 2010s, first evidence in rodent models suggested promising antitumor action of gas plasma technology. Medical gas plasma is a partially ionized gas depositing multiple physico-chemical effectors onto tissues, especially reactive oxygen and nitrogen species (ROS/RNS). Today, an evergrowing body of experimental evidence suggests multifaceted roles of medical gas plasma-derived therapeutic ROS/RNS in targeting cancer alone or in combination with oncological treatment schemes such as ionizing radiation, chemotherapy, and immunotherapy. Intriguingly, gas plasma technology was recently unraveled to have an immunological dimension by inducing immunogenic cell death, which could ultimately promote existing cancer immunotherapies via in situ or autologous tumor vaccine schemes. Together with first clinical evidence reporting beneficial effects in cancer patients following gas plasma therapy, it is time to summarize the main concepts along with the chances and limitations of medical gas plasma onco-therapy from a biological, immunological, clinical, and technological point of view.

## 1 Introduction

The idea of using gas plasmas in medicine and its first application in patients dates back to the early 20th century [1]. In those times, several technologies were invented and explored for beneficial medical effects, such as radiotherapy [2] and photodynamic therapy [3]. Yet, it was not until the early 21st century for gas plasma technology to be re-discovered and tested for its therapeutic potential in medical conditions. The first randomized clinical trial in this field, coined plasma medicine*,* was reported in 2010 on antimicrobial effects in the context of wound healing [4]. Based on further experimental and clinical evidence, from 2013 onwards, several gas plasmas devices were approved as medical device class IIa in Germany and Europe for treating chronic wounds, ulcers, and other skin conditions linked to infections [5]. With the medical device regulation (MDR) update in the EU and renewed approval procedures, some medical gas plasma devices are ranked as class IIb device today. It is important to mention that this approval allows unrestricted application of this technology within the mentioned indications, e.g., support of wound healing and treatment of infected skin and skin appendices. Hence, today, many thousands of patients in dermatology have benefited from gas plasma therapy showing a superior safety profile with virtually no side effects or long-term off-target consequences reported in the literature [6] while being well-tolerated and easy to apply.

In parallel to gas plasma research in disinfection and wound healing, other fields of applications were explored, including oncology [7]. Apart from earlier rudimentary *in vitro* reports, the first two back-to-back studies suggesting medical gas plasma anticancer activity rodent tumor models were published [8] and accepted [9] in 2010. Shortly after, the same French researcher consortium was the first to demonstrate the combined efficacy of gas plasma exposure with established cytostatic anticancer drugs in a mouse model of pancreatic cancer [10]. These and other studies propelled the idea of studying the possibility of employing gas plasma technology for various types of cancer.

This review at hand summarizes the main principles of medical gas plasmas. This includes their biological consequences in cells and tissues, the question of selectivity, their role of immuno-stimulation, and pre-clinical and clinical evidence on gas plasma cancer treatment. It will also outline challenges and opportunities of how gas plasma technology could be engaged within clinical oncology by complementing existing treatment modalities such as checkpoint immunotherapy.

## 2 (Medical) gas plasma

### 2.1 Background

Physical plasma, the so-called fourth state of matter, is a (partially) ionized gas. The interested reader is referred to text books on plasma physics [11] and chemistry [12] for deeper insight into physico-chemical plasma processes. In brief, physical plasmas are gases that are energized until electrons become energetic enough to dissociate from atoms, forming free electrons and ions. This forms the plasma state, which has distinct properties from gases, including, e.g., a higher conductivity (but overall electric neutrality), in some cases a larger degree of compactness (i.e., more molecules per volume unit), and a non-equilibrium state. Usually, gas plasmas are formed at high temperatures or at low pressure. Technological leap innovations in the late 1990s and 2000s and medically-compliant plasma source design allowed the generation of non-thermal (‘cold’) atmospheric pressure (ambient air) physical (gas) plasmas for medical application. Only a few gas plasma sources are approved for applications in patients to treat medical conditions, while the large majority of experimental or prototype gas plasma systems reported in the literature are not allowed for patient treatment due to the lack of medical device approval based on patient studies and electric or toxicity safety profiling. Apart from that, an array of relatively low-priced plasma sources is available for cosmetic purposes and home care use. Those sources are often hot, cauterize the skin, and are not approved to treat medical conditions. In the present work, no distinction will be made regarding cancer research results obtained with approved vs. non-approved gas plasma sources since, currently, none of the approved gas plasma devices in dermatology have received an indication extension towards oncology. This means gas plasma cancer treatment is still in its experimental, exploratory state. Noteworthy, there are also non-tissue-preserving (thermal, necrotizing) gas plasma processes used in medicine for tissue cutting (plasma scalpel, also called thermal knife) and hemostasis (blood coagulation by thermal sealing) [13,14]. These plasmas acting on tissues primarily via thermal effects are not the topic of this review in contrast to the gas plasma devices operated at body temperature.

There are different geometries of gas plasma devices. From the medical perspective, the perhaps biggest difference is i) whether the device needs an external feed gas, such as the noble gas argon (plasma jets), or not, and ii) if the device has a flat, coin-like surface shape for large-area treatments (e.g., surface, volume, or floating-electrode dielectric barrier discharges, DBDs) [15] or a fine plasma plum like a cold flame that is extending from a usually pen-like device which can be operated like a scalpel to facilitate high precision treatment and having superior cavity penetration ability for uneven body surface topologies (plasma jets) [16]. An extra gas supply (usually: plasma jets; sometimes: DBDs) standardizes the gas plasma ignition process while producing additional gas costs and leading to less flexibility in terms of operation needs. A flat surface (some DBDs) helps for large-area applications, such as extensive leg ulcers seen in diabetes patients [17], but covers the view of the area to be treated and plasma-exposes the entire area at the same intensity. By contrast, plasma jets – in addition to the ability of changing the treatment height based on the given surface topology – can be directed to some treatment areas for longer durations than others, depending on the clinician’s need. For example, a group of palliative head and neck cancer patients received gas plasma jet therapy [18], where some tumor areas were plasma-treated longer than others (personal communication Hans-Robert Metelmann).

### 2.2 Modes of action

**Fig. 1:**
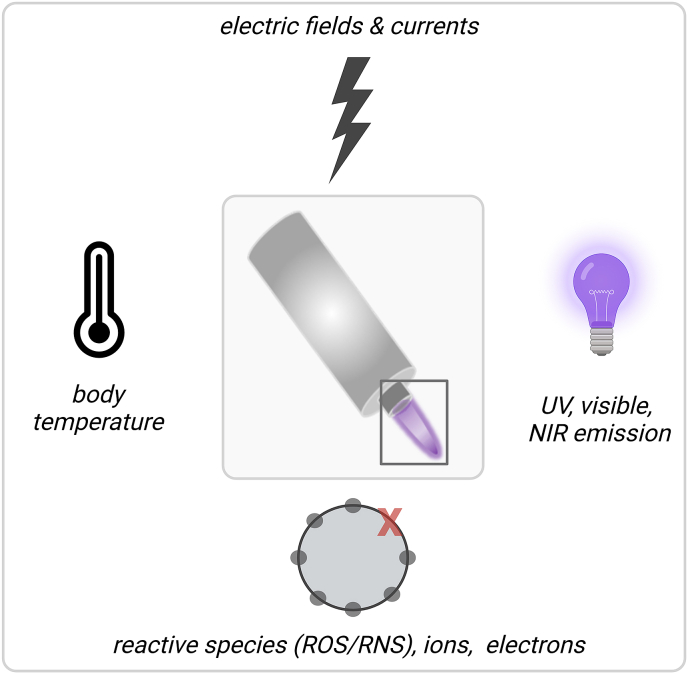
Gas plasma properties representative of a gas plasma jet device. UV: ultraviolet; NIR: near infrared; ROS: reactive oxygen species; RNS: reactive nitrogen species.

**Fig. 2:**
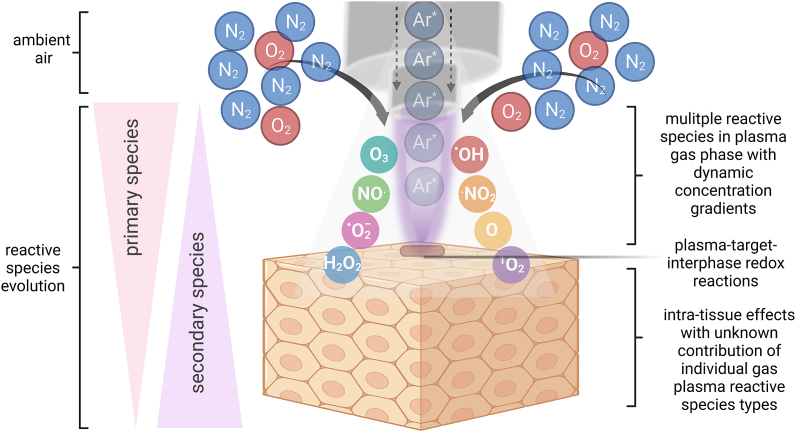
Principle of reactive species generation in the case of an atmospheric pressure argon plasma jet. Argon gas is partially ionized in the head of the jet and driven out to the ambient air by the pressure of the argon gas. Ambient air O2 and N2 react with argon radicals and metastables, generating several reactive oxygen and nitrogen species simultaneously, such as O3,.OH,.NO2, O, 1O2, H2O2, NO., and.O2-. The reactive species densities are not fixed but change in mixture and individual concentration from over the jet nozzle release point to the target, such as tissue. The plasma gas phase and relatively-well characterized while the reactive species chemistry at the plasma gas-target interphase and below has not been elucidated sufficiently due to the lack of specific tracers unavailable for many reactive species types.

**Fig. 3:**
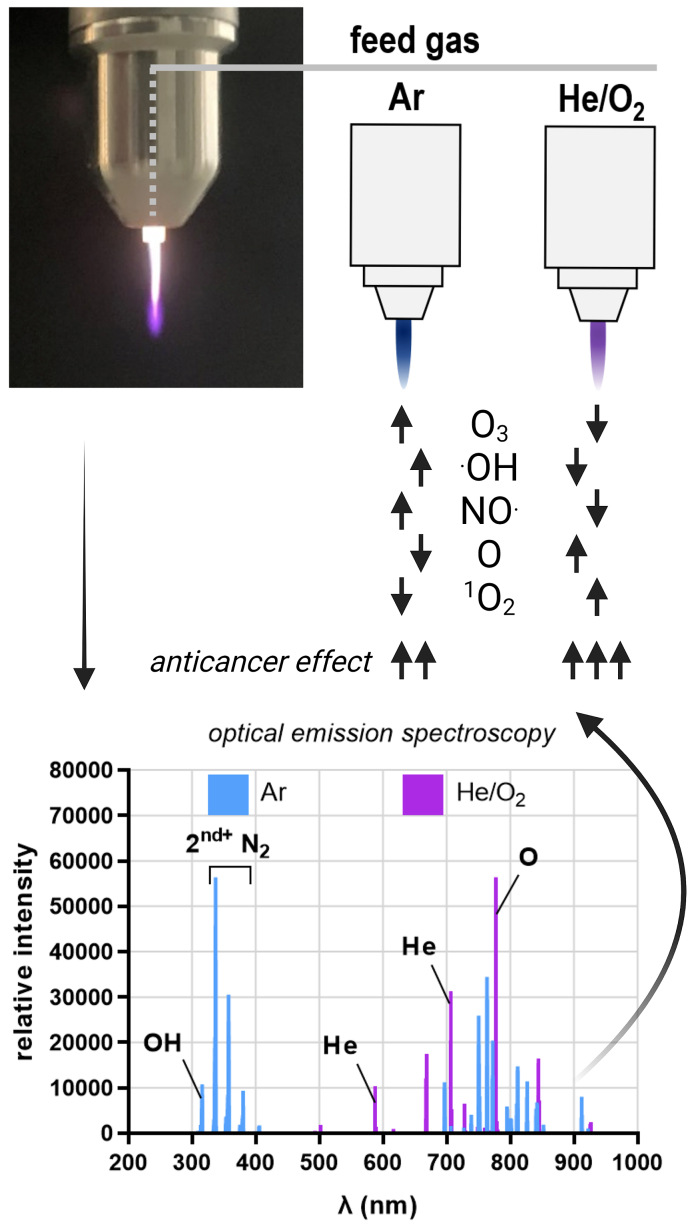
Principle of reactive species tuning at the example of the kINPen atmospheric pressure plasma jet. Its standard operation gas is pure argon, which leads to a distinct reactive species profile in the plasma gas phase (optical emission spectroscopy, as shown below, and other plasma diagnostics) and in treated liquids, including O3,.OH, and NO.. If the jet is operated with He/O2, the relative level of such species declines, while the generation of O and 1O2 is more pronounced. While the kINPen argon operation mode already poses profound anticancer effects, the He/O2 operation increases such effect even more, which is likely a result of the altered reactive species mixture. Adapted from Refs. [37,132].

Gas plasmas are multicomponent systems, which is due to the intrinsic nature of the physical plasma having multiple characteristics, such as the generation of electric fields, the light emission in the UV up to the infrared range, the production of electrons and ions, as well as the secondary generation of reactive oxygen and nitrogen species (ROS/RNS) when discharged into ambient air (Fig. 1). The term ROS will be used to cover both ROS/RNS in this review, as RNS mostly contain reactive oxygen, too [19]. It is generally accepted that gas plasmas primarily act on cells via ROS deposition, which induces oxidative eustress or distress [20]. The former is defined as a surplus presence of ROS that is, however, low enough to act as a cellular regulator by inducing redox signaling processes, usually via thiol switches [21]. By contrast, the latter describes effects of ROS concentrations beyond signaling functions, such as cell death induction via ferroptosis or necroptosis [22,23] and non-reversible oxidative modifications of proteins and lipids [24,25]. This ultimately puts the field of plasma medicine in the realm of applied redox medicine [26]. While it is understood that in redox biology the generalized mention of ROS often is discouraged towards mentioning individual reactive species types, the term ROS is suitable in the field of gas plasma biomedical sciences because here, there is always a generation of multiple reactive species in the gas phase (Table 1). With atmospheric pressure plasma jets, for instance, reactive species generation is achieved by feeding a noble gas into the device. The gas is then partially ionized by a high-voltage electrode. The ionized noble gas species are then expelled to form ROS, such as O_3_, ^.^OH, ^.^NO_2_, O, ^1^O_2_, H_2_O_2_, NO^.^, and ^.^O_2_^-^ (Fig. 2). In the plasma gas phase, the types of species are relative well characterized, especially for widely used devices such as the kINPen [27]. In the gas plasma-tissue interphase and within tissues, however, there is a lack of tools in redox biology to unambiguously dissect the contribution of each reactive species to the effect observed, including questions on penetration depths. It can be assumed, however, that the half-lives given for selected reactive species types in buffer (Table 1) correlate with their penetration depth in tissues, but experimental data for gas plasma on this hypothesis are lacking. For some components, such as H_2_O_2_, it has been recently described that its diffusion range in tissue (brain) is expected to be up to 100 μm [28]. One way of addressing the issue of the individual species’ contribution is operating plasma jets with different feed gases that subsequently generate distinctly different reactive species mixtures. For instance, the kINPen shows high levels of, e.g., O_3_, ^.^OH, and NO^.^ when operated with Ar, while in He/O_2_ operation, a pronounced production of O and ^1^O_2_ is observed (Fig. 3). Similar findings in enhanced cytotoxic activity were made for two other plasma jets when comparing He to He/O_2_ operation [29,30], suggesting a general principle based on increased toxicity with increased O and ^1^O_2_ levels. However, it should be noted that for feed gas alterations there are also other changes in the plasma, such as electron densities, electric fields (leakage current), and levels of argon or helium metastables with unknown effects in (redox) biology, which could contribute to the oxidative stress responses observed. The idea of oxidative eustress and distress is also reflected in the concept of hormesis [31,32]. Hormetic actions are observed when a given agent at a low concentration performs (partially) opposite actions than at high concentrations, for which several examples exist in pharmacology [33,34]. With regard to ROS in general and plasma medicine in particular, it has been well described that low ROS levels promote, e.g., wound healing, while higher levels are, for instance, tumor-toxic. This explains the pleiotropic effects of gas plasma treatment in biomedical applications ranging from stimulation to destruction [19], depending on the energy or exposure time applied, which often correlates with ROS deposition in a linear manner [35]. As a practical example, the gas plasma jet treatment time (applied in several sessions throughout 12 days) required to promote wound healing in a mouse model was 5s, while the 20s of treatment already abrogated some of the positive effects [36]. Using the same plasma setup, the gas plasma treatment time required to observe anti-melanoma effects (implanted subcutaneously to mice) was 240s [37]. These figures illustrate the importance of considering the exposure time (or ‘dose’) and hormetic effects when it comes to gas plasma cancer treatment.

**Table 1:** Non-exhaustive list of primary or secondary reactive species and oxidants identified (or in the plasma gas phase: computer modeled) to be generated by gas plasma devices. If possible, at least three different devices were referenced for each type of reactive species and phase.

name (approx. half-life in buffer)	abbreviation	plasma gas phase	plasma-treated matrices
atomic oxygen (unknown)	O	[[305], [306], [307], [308]]	[[309], [310], [311]]
hydroperoxyl radical (10^0^ s [312])	HO^.^_2_	[313]	–
hydrogen peroxide (stable)	H_2_O_2_	[314,315]	[35,139,142]
hydroxyl radical (10^-9^ s [316])	^.^OH	[[317], [318], [319]]	[40,137,320]
hypochlorous acid (10^2^ s [321])	OHCl	–	[30,169,322]
metastables (e.g., Ar*, N_2_(C), He_m_*) (unknown)	Ar*	[[323], [324], [325]]	–
nitric oxide (10^1^ [326])	NO^.^	[[327], [328], [329]]	[320,330,331]
nitrogen dioxide (10^-5^ [332])	^.^NO_2_	[[333], [334], [335]]	[274]
nitrate (stable)	NO_3_^-^	HNO_3_ [[336], [337], [338]]	[[339], [340], [341]]
nitrite (stable [342])	NO_2_^-^	HNO_2_ [[343], [344], [345]]	[[346], [347], [348]]
ozone (10^1^ [349])	O_3_	[350,351]	[[352], [353], [354]]
peroxynitrite (10^0^ [355])	ONOO^-^	[356,357]	[165,344,358]
singlet oxygen (10^-5^ s [359])	^1^O_2_	[29,40,360]	[30,296,361]
superoxide anion (10^-6^ s [362])	^.^O_2_^-^	–	[[363], [364], [365]]

Two ways of utilizing gas plasmas in biomedicine and against cancers have been explored in the past decade. The first is the direct application of gas plasmas onto the target; the second is the production of gas plasma-treated liquids (PTL). Direct applications are performed, as the name says, with the gas plasma device in direct contact with the target (direct conductive treatment) or – especially in the case of self-ignited plasma jets – with the gas plasma device floating above the target (remote direct treatment). In the former situation, the treated target serves as a third electrode, attracting all gas plasma components by forming a physical plasma channel between the device towards the target. Consequently, several physical effectors, such as UV light, direct thermal transfer, immediate exposure to electrons and ions, the ROS generation directly on the gas plasma-tissue interphase, and supply of electric fields. Disentangling the individual effects of all these components is challenging without manipulating the gas plasma ignition itself (e.g., adding grounding targets or quartz classes). Hence, there is lack of methods to distinguish the partially overlapping consequences of such effectors within any type of tissue. When investigating cells submerged in excess amounts of liquids, such as in *in vitro* experiments, it becomes clear that ROS are responsible for most effects observed [19]. To what extent this is also the case for tissues remains to be established. In the case of remote direct gas plasma exposure, especially with plasma jets, a ‘cloud’ of ROS is being expelled towards the target tissue, while the other effectors are transported to a minor extent or not at all. If the plasma jet does not ‘touch’ the target, no flow of electrons from the device is established, electric fields remain mostly confined to the plasma jet, and UV light is already partially absorbed in the air. Also, there is a change in ROS composition, as ROS types and concentrations are a function of the position perpendicular to the origin of gas plasma generation [[38], [39], [40], [41]]. The further away, the less complex the ROS mixture is and the more it is dominated by a few, long-lived reactive species, such as, e.g., NOx and ozone [42]. Therefore, it is unsurprising that conductive gas plasma cancer treatment is more effective than the remote counterpart in cellular 3D biopolymer models and in vascularized *in ovo* tumor organoids [43]. With regard to gas plasma-treated liquids (PTL), studies on its anticancer actions have been extensively reviewed recently [44]. Main conclusions are summarized in Box 1.

Box 1 **Main principles and mechanisms of medical gas plasma technology.**

⁃ Medical gas plasma technology is a partially ionized gas operated at body temperature
⁃ Gas plasma-induced biological responses are majorly driven by ROS
⁃ The field of (medical gas) plasma medicine is part of the field of Applied Redox Medicine
⁃ Only a few gas plasma devices are approved as class IIa/IIb medical device for the treatment of medical conditions in the EU
⁃ Approved gas plasma indications in dermatology are related to wound healing and infection, oncology applications are still experimental
⁃ The safety profile of gas plasma treatment in patients is excellent, with no adverse events or major side effects reported so far

Alt-text: Box 1

## 3 Experimental gas plasma cancer treatment and immunogenicity

### 3.1 In vitro modes of cell death and mechanisms

In this work, there will be no distinction made on the differences between, e.g., gas plasma source types, working principles, or feed gas compositions, in order to focus on common principles. Many *in vitro* reports on the inactivation of cancer cells using gas plasmas have been filed in the last few years. The majority have identified gas plasma-treated tumor cells to succumb to regulated cell death, a category with more than a dozen of family members [45]. Besides apoptosis, which is mostly reported, a few studies claim to have also identified other types of cell death, albeit appropriate controls are often missing to unambiguously identify one cell death modality over another, besides overlap existing between cell death modalities. In gas plasma-treated cancer cells, ferroptosis [[46], [47], [48], [49]], lysosomal cell death [50,51], autophagy-related cell death [[51], [52], [53]], pyroptosis [54], and necrosis [[55], [56], [57]] were reported. Necrosis, however, can be easily achieved in any type of cancer cell monolayer directly exposed to the gas plasma without a minimal layer of liquid above either due to drying effects of the feed gas pressure or due to electric field effects if the monolayer (or sometimes a metal plate below the cell culture plate) is used as a counter electrode. The *in vivo* relevance of these mechanisms is, however, likely limited [58]. With regard to apoptosis, all typical hallmarks of apoptotic cell death were identified in tumor cells (if the cell line in question was still capable of full-spectrum apoptosis induction), such as activation of caspases 3 and 7 [59], loss of the mitochondrial membrane potential [60] and subsequent augmentation of ^.^O_2_^-^ production [61], outer membrane blebbing, cytochrome c [62] and lactate dehydrogenase release [54], decreased intracellular ATP [63] and lactate [64] production, nuclear fragmentation [65], phosphorylation of the histone 2.AX [66], and an increase in the Bax/Bcl2 ratio [67], together with a post-incubation time-dependent increase of externalized phosphatidylserine [68]. These results are strengthened by studies showing that pre-incubation with the pan-caspase inhibitor Z-VAD-FMK abrogates gas plasma-induced cancer cell apoptosis *in vitro* [54,[69], [70], [71], [72], [73], [74]]. By contrast, a set of authors consistently reports a lack of cancer apoptosis abrogation by Z-VAD-FMK pre-incubation [[75], [76], [77], [78]], which could be either due to lacking control experiments (with, e.g., staurosporine) that the Z-VAD-FMK agent was functional, or, on what the authors also speculate, due to the cell lines investigated. The authors did find activated caspase 3 in their studies, but it should notwithstanding be noted that there is also evidence of caspase-independent apoptosis, called oxeiptosis [79,80]. Besides cell death, two studies have implicated a role of cellular senescence in gas plasma-treated cancer cells [81,82].

Many reports provided considerable evidence that gas plasma-induced regulated cell death can be abrogated by adding antioxidant enzymes or antioxidants [49,70,[83], [84], [85], [86], [87], [88], [89], [90], [91], [92]]. In addition, virtually all cell lines, regardless of their malignant or non-malignant origin, have been reported to respond to gas plasma exposure, including cytotoxic effects at increased doses or treatment times. This is best exemplified in a recent screening of 36 tumor cell lines and two non-malignant cell types, showing an almost hundred-fold difference in sensitivity between the most sensitive and resistant tumor cell lines, while the non-malignant cell types showed modest sensitivity [93]. Although not directly included in this study, cross-comparison of the sensitivity of primary lymphocytes using the same gas plasma jet reveal these cells to be the most sensitive in the cell type screening, and highly more sensitive than their leukemic counterparts [94,95]. These and other findings [96,97] support the notion that gas plasma-induced cell death is not a prerequisite of the malignancy grade of the cell type in question but must lie in its ability to cope with oxidative stress and translate it into cell death signaling. This idea contrasts the claims of studies that had investigated selective actions of gas plasma-induced cancer cell toxicity [[98], [99], [100], [101], [102], [103], [104], [105], [106], [107], [108], [109], [110], [111], [112], [113], [114], [115], [116], [117], [118], [119], [120]]. In addition, two former dogmas of either membrane-based aquaporin [121] or catalase and NOX expression [122] dictating tumor cell sensitivity to gas plasma-induced cell death were not supported by that recent study screening 36 cell lines [93]. By contrast, other processes, such as metabolic activity and redox-related signaling, may be more critical in gas plasma-induced cancer cell death. Moreover, a recent tumor cell line comparison using the same cell lines revealed a key role of cell cycle-related genes associated with sensitivity to H_2_O_2_-induced cell inactivation [123]. A the same time, in acute myeloid leukemia (AML), it is known that compared to non-malignant myeloid cells, AML cells can cope with higher ROS levels while showing less cell death following ROS exposure, which is due to mutations in p38-MAPK-induced cell death signaling pathways, ultimately hampering apoptosis induction [124]. Four lessons can be learned from these findings. First, the dominant cell death modality in gas plasma cancer treatment is regulated cell death within the existing modalities already described in the literature. Second, this cell death strongly depends on gas plasma-generated ROS and their deposition into liquids *in vitro*. Third, these two findings are consistent across many dozen different gas plasma devices reported on, regardless of the different scales or energies needed to produce the anticancer effect (i.e., some devices will need seconds, other minutes, depending on the gas plasma device geometries, feed gas fluxes, etc.). Fourth, with sensitivities of tumor cells to succumb to gas plasma-induced cell death at up to 100-fold differences (and non-malignant, healthy cells lying in between), it becomes evident that an overarching anticancer selectivity of gas plasma treatment cannot be claimed, at least *in vitro*. Selectivity, however, is of less important in local treatment modalities, as can be seen further below.

**Fig. 4:**
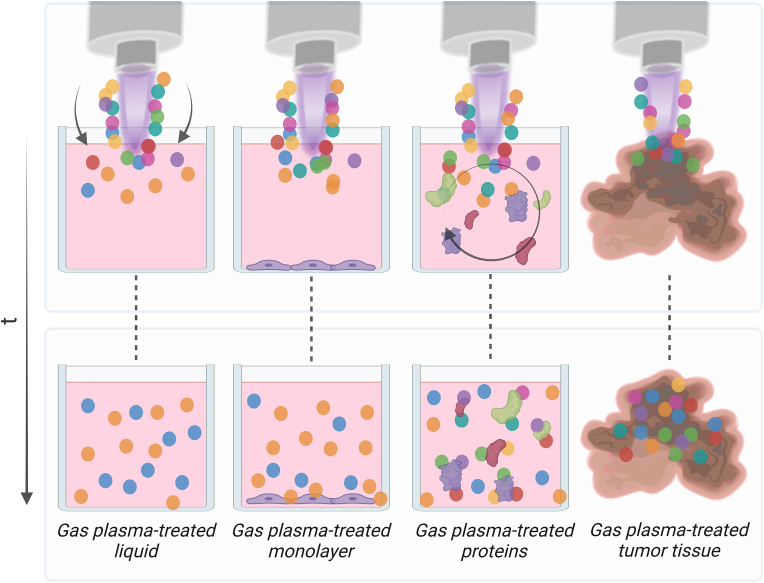
Despite deposition of similar short-lived reactive species mixes, liquid-dominated *in vitro* systems showed effects prevailed by long-lived oxidants due to long diffusion distances needed to the monolayers or long time differences from production to the application, as the case with gas plasma-treated liquids. By contrast, highly concentrated proteins in combination with feed-gas induced vectors may lead to the transport of smaller targets, such as proteins, close to the gas plasma-liquid interface with the highest concentration of short-lived species, ultimately leading to unique patterns of oxidative post-translational protein modifications (oxPTMs). In the case of tissues, such as ulcerating skin cancer, abundant biomolecules react with the gas plasma-derived reactive species to become oxidized, leading to signaling responses and tumor cell death.

Hundreds of chemical redox reactions occur in the plasma gas phase [125] before it hits the target. *In vitro*, the target is mostly a liquid in which the cancer cells are submerged into. Albeit it is undisputed that a range of primary gas plasma-derived species initially diffuse into the plasma-liquid interphase [126], it is also clear that the primary trait of these ROS is their reactive and short-lived nature, ultimately yielding half-lives and diffusion distances often too short for reaching the cells directly (Fig. 4). For example, ^.^OH have a diffusion distance in physiological buffer of about 0.1μm; ONOO^-^of about 50μm [127]. Even if the supernatant of a cell monolayer is taken off to decrease penetration distances of gas plasma-derived ROS to cancer cells, these figures illustrate that also in these cases, the majority of reactive species will have reacted to other, less active species. These species are H_2_O_2_, NO_2_^-^, and NO_3_^-^ in the case of most plasma sources [83,[128], [129], [130], [131]]. In a second scenario, using dedicated feed gas conditions, the amplified production (or reduced scavenging by O_2_) of O and ^1^O_2_ facilitates the generation of HOCl from Cl^-^ in the liquid [29,132]. HOCl essentially consumes H_2_O_2_, leading to two distinct gas plasmas cancer treatment scenarios – H_2_O_2_-dominated vs. HOCl-dominated – that have been shown to be decisive in the anticancer capacity of gas plasma in specific cancer cell types *in vitro* and *in vivo* [30,37]. Both agents are stable enough also to reach cancer cells at longer distances. There is also evidence of ONOO^-^-dominated chemistries, which, however, are thought to be more important in antimicrobial gas plasma effects [[133], [134], [135]]. As mentioned above, it is also rather short-lived and appears in only low concentrations in the nanomolar to micromolar range under cell culture conditions following gas plasma exposure [128,136]. The dominant effect of ROS on gas plasma-mediated anticancer effects *in vitro* is best demonstrated by the abrogation of cytotoxic effects using antioxidants or antioxidant enzymes. For instance, n-acetyl-cysteine (NAC), a pan-ROS scavenger, efficiently protects cells from cytotoxic effects in both the H_2_O_2_ and HOCl-dominated gas plasmas [30,69,73,83,116,137,138]. Intriguingly, catalase, a H_2_O_2_-degrading enzyme, near-fully abrogates gas plasma-induced toxicity in several gas plasma devices and cell types tested so far in H_2_O_2_- [35,69,85,128,[139], [140], [141], [142], [143], [144], [145], [146], [147], [148], [149], [150], [151], [152], [153], [154], [155]] but not HOCl-dominated gas plasma or PTL regimens [29,30] and irrespective of the malignant or non-malignant nature of cells investigated. Several reports show that the experimental addition of equimolar concentrations of H_2_O_2_ does not recapitulate all of the effects observed with gas plasma exposure or PTL [85,142,151,156], which is likely due to errors in concentration synchronization, antioxidants in media and time and kinetic effects, and/or differences in application (bolus addition of high concentration of chemical H_2_O_2_ vs. continuous production of H_2_O_2_ in gas plasma-treated liquids). Hence, H_2_O_2_ is a major contributor to liquid-rich gas plasma anticancer applications, including PTL. Equivalent effects were shown *in vitro* and *in vivo* for the approved gas plasma medical device kINPen and phosphate-buffered saline, 0.9% sodium chloride, Ringer’s lactate, and cell culture media [85,128,142,148], suggesting rather minor roles of agents other than H_2_O_2_. These results were confirmed for other gas plasma devices [157]. Two groups report non-protective roles of catalase in gas plasma-treated Ringer’s lactate [154,158], which could be due to its addition prior to and destruction via the gas plasma treatment in their studies, which effectively inactivates catalase and inhibits its H_2_O_2_ removal capacity. It should also be noted that there are several studies providing evidence of a combined effect of (gas plasma-derived) H_2_O_2_ with NO_2_^-^ in the form of PTL for anticancer treatment [[159], [160], [161]]. Even if this was the case, the gas plasma process would be dispensable compared to the economically favorable chemical production and use of such species at pharmacological precision. Regarding the type of liquid investigated as PTL, it must be emphasized that clinically approved liquids such as 0.9% sodium chloride and Ringer’s lactate need to be tested. At the same time, the complex formulations of cell culture media are unsuitable for approval and routine clinical application [162].

### 3.2 In vivo cancer models

Several types of gas plasma devices have been successfully employed to facilitate antitumor removal *in vivo* (Table 2). With a few exceptions, most models were murine, and the majority were xenograft, i.e., a non-murine tumor was implanted in immunodeficient mice, in contrast to syngeneic models that allow studying antitumor immunity in addition to tumor growth. Moreover, tumors can be implanted at convenient (i.e., easy to reach), non-orthotopic sites (e.g., subcutaneous ovarian cancer), or at locations where they occur naturally (orthotopic, e.g., intraperitoneal colorectal carcinomatosis). There have been several reviews on specific tumor entities and different gas plasma devices, and besides an array of specific details, several overarching conclusions can be reached. First, gas plasma-treated medically approved liquids are suitable for efficiently treating peritoneal carcinomatosis [162]. At the same time, it is convincingly shown that similar effects can be reached with chemical H_2_O_2_ at similar concentrations [142], so this specific application is expected to be of minor future clinical relevance and, perhaps, interest. Second, *in vivo*, skin cancer models, especially melanoma, have been investigated most abundantly, and besides tumor-toxic, also pro-immunogenic effects (see below) were observed. However, it needs to be noted that these transcutaneous gas plasma treatments (the melanoma is usually injected subcutaneously) are only efficient in mice due to their thin skin; such gas plasma treatment likely does not work in the case of human intradermal melanoma metastases [163]. Third, one notable study found significantly reduced tumor recurrence after sub-complete tumor excision followed by intra-surgical gas plasma exposure of the surgical wound [164] (see next paragraph). Similarly, gas plasma-treated, ROS-enriched biopolymer scaffold/hydrogel was reported to promote intra-surgical remission-free outcomes in mice [165]. Fourth, a report with exceptional comprehension (>400 animals treated and investigated in parallel over 1 year) tested the monthly gas plasma exposure of the murine oral mucosa over 12 months with two independent argon gas plasma jets and in presence and absence of a carcinogen promoting oral squamous cell carcinoma (SCC) [166]. Besides the fact that gas plasma did not induce any type of lesion or SCC (i.e., it is not mutagenic and safe to be applied multiple times to mucosa tissue), it appeared that gas plasma treatment increased the number of tumor-free mice exposed to high carcinogenic doses, introducing the idea of gas plasma exhibiting a potential tumor-protective effect in terms of cancer prevention. This notion is underlined by a study investigating spontaneous UV-induced cutaneous SCC following a clinically approved gas plasma treatment (kINPen MED), finding significant suppression of SCC features in mice [167]. However, many *in vivo* studies were performed with experimental and not certified gas plasma devices. While this creates a certain diversity in approaches and device designs, it limits clinical translation. Therefore, the testing of clinically approved gas plasma devices would potentially help clinicians and oncologists in their decision of whether to test the technology in selected individual cases in, e.g., oncology. Such clinical gas plasma testing is the main bottleneck to advance the field as of now, and using safe and known technology would help mitigate this circumstance. In addition, future studies should focus on orthotopic tumor models rather than injecting all tumors types mostly subcutaneously. Albeit orthotopic models are more challenging to work with, the translational value of the results can be greater.

**Table 2:** Non-exhaustive overview of tumor entities and their growth successfully being decreased in experimental mouse models using direct gas plasma treatment or exposure to gas plasma-treated liquids (PTL).

entity	genetics	location	gas plasma	application	cell lines	refs.
bladder	xenograft	non-orthotopic	direct plasma, PTL, plasma-treated hydrogel	tumor-therapeutic	SCaBER, T24, J82	[110,165,366]
breast	syngeneic, xenograft	orthotopic, non-orthotopic	direct plasma, PTL	tumor-therapeutic, anticancer immunity	4T1, MCF7, MDA-MB-231	[164,173,[367], [368], [369]]
CNS (glioblastoma)	syngeneic, xenograft	orthotopic, non-orthotopic	direct plasma, PTL	tumor-therapeutic	U87MG, SB28	[174,186,[370], [371], [372]]
colorectal	syngeneic, xenograft	orthotopic, non-orthotopic	direct plasma, PTL	tumor-therapeutic, anticancer immunity, vaccination	CT26, COLO 205	[85,142,171,[373], [374], [375], [376]]
gastric	xenograft	orthotopic	PTL	tumor-therapeutic	GCIY	[377]
gynecological (cervical, ovarian)	xenograft	non-orthotopic	direct plasma, PTL	tumor-therapeutic	HEC-1, NOS2, SiHa, ES2	[158,[378], [379], [380], [381], [382], [383]]
leukemia	xenograft	non-orthotopic	direct plasma	tumor-therapeutic	Ba/F3 BCR-ABL1	[72]
lung	xenograft	non-orthotopic	direct plasma	tumor-therapeutic	A549	[384]
melanoma	syngeneic, xenograft	orthotopic	direct plasma, PTL	tumor-therapeutic, anticancer immunity, vaccination	B16, A375	[37,65,98,110,132,136,149,164,183,189,256,258,[299], [300], [301], [302], [303], [304],[385], [386], [387]]
muscle (myosarcoma)	syngeneic	non-orthotopic	direct plasma	tumor-therapeutic, anticancer immunity	MX-7	[177]
neuroblastoma	syngeneic	non-orthotopic	direct plasma	tumor-therapeutic	Neuro2a	[388]
pancreas	syngeneic, xenograft	orthotopic, non-orthotopic	direct plasma, PTL	tumor-therapeutic, anticancer immunity	PDA6606, MiaPaCa-2, Capan-2, AsPC-1	[10,86,90,389]
cutaneous/oral squamous cell carcinoma (SCC)	UV-induced, DBP-induced, xenograft	orthotopic	direct	tumor-therapeutic	A431, FaDu, SCC15	[166,167,258,390,391]

Chemical, physical, and pharmacological agents can also promote an immuno-stimulating type of cell death called immunogenic cell death (ICD) [168]. ICD has several hallmarks, which can be tested to a limited extent *in vitro*, and gas plasma-induced ICD features such as ATP release [[169], [170], [171]], externalization of calreticulin (CRT) [37,85,142,143,169,[172], [173], [174], [175]], HMBG1 translocation [85,169,176,177], and dendritic cell (DCs) stimulation [142,169] identified *in vitro* motivated first *in vivo* investigations. These gas plasma-induced ICD processes are hypothesized to support the activation of DCs to present tumor antigens for T-cell priming in draining lymph nodes to elicit the cancer-immunity cycle [178], as described in previous concepts [179]. Accordingly, ICD elements were found in gas plasma-treated tumors, for instance, increased CRT and HMGB1 in murine tumors [171,173,180]. In addition, markedly increased numbers of professional phagocytes and effector T-cells were found in gas plasma-treated tumor tissues [37,171,175]. Critically, the gold standard model for validating *in vivo* ICD responses is vaccination with *in vitro*-killed tumor cells, followed by an incubation period for mounting an antitumor tumor immune responses, a re-challenge with live tumor cells being injected, and assessment of the number of tumors grown [181]. Two studies, one with a medically approved gas plasma device, provided evidence of *bona fide* ICD induction using gas plasma technology [37,136]. Even more, one recent study was the first to provide evidence and mechanisms on true abscopal effects of gas plasma exposure in antitumor remission [173], i.e., an immune-related reduction of a non-treated tumor in mice harboring a second tumor that was exposed to the treatment (gas plasma) in question. Moreover, combination treatment with antibodies supporting antitumor-immune responses, so-called immune-checkpoint blockade antibodies [182], augmented gas plasma cancer treatment in a melanoma mouse model [183]. Finally, that same group provided stunning evidence that gas plasma technology might be a suitable adjuvant treatment in oncological surgery. Specifically, the authors had grown tumors in mice and incompletely removed them surgically. Next, the tumor wound was gas plasma-treated, and the wound was closed. Subsequently, tumors were allowed to regrow, and the tumor microenvironment (TME) was analyzed for immuno-infiltrates. Both tumor progression and re-growth were reduced in the group of mice that had received gas plasma treatment, while intra-tumoral leukocyte numbers were elevated, suggesting in situ ICD induction [164]. These studies already contain important pre-clinical elements, which could and should be considered in adjuvant tumor oncology. There are several addressable experimental needs to support such considerations, however. First, more *in vivo* lymphocyte depletion experiments are needed to reinvigorate the role and necessity of T-cells in gas plasma-promoted antitumor immunity as common in onco-immunological schemes. Second, most tumor models investigated so far are too aggressive for long-term observations (if synchronized sacrifice dates of untreated controls are warranted for comparable TME investigations). Testing more durable, less aggressive tumor models is needed to identify potential adaption responses to gas plasma exposure and the long-term durability of induced antitumor immune responses. Third, as customs in to the age of cancer immunotherapies, more studies should focus on syngeneic cancer models instead of using the often-used adaptive immunity-deficient xenograft tumor models. Fourth, there is a great need to combine gas plasma treatment with treatments approved for the cancer entity in question to reveal how plasma technology may add as an adjuvant to existing oncological radio-, chemo-, targeted, and immunotherapeutic routines. Among the existing six *in vivo* gas plasma combination treatment studies are those using gemcitabine [184] for pancreatic cancer [10], temozolomide [185] for glioblastoma [186], dacarbazine [187] for melanoma [98], electrochemotherapy [188] for melanoma [189], imiquimod [190] for melanoma [37], and anti-PD1 checkpoint therapy [191] for melanoma [183]. There is no *in vivo* report on combining gas plasma exposure with radiotherapy or targeted therapy yet, besides lacking *in vivo* combination data on drugs heavily used in oncology, such as platinum-based chemotherapeutics. The same is true for checkpoint antibody immunotherapies apart from anti-PD-1.

## 4 Clinical gas plasma cancer treatment

To date, no clinical routine has been established for any type of malignancy involving cold gas plasma treatment. ‘Cold’ is emphasized because the primary engineering goal of those gas plasma technologies was to be compatible with tissues, e.g., for the partially ionized gas to be cold enough to be applied directly to tissues without burning or necrosis [6]. By contrast, the hotter plasma pendant (argon plasma) electrosurgery/electrocauterization is frequently used during tumor surgery to cut tissue and cauterize and necrotize blood vessels to facilitate hemostasis [192,193]. Such hot devices are sometimes also called by names known in ‘cold’ plasma medicine, such as Neutral Argon Plasma or PlasmaJet (NCT02376231, NCT049004042, NCT01596985, NCT04355312). In turn, cold gas plasma treatment of patient-derived tumor tissues elicits apoptosis [[194], [195], [196]]. Some companies marketing these electrosurgical devices started a few years ago to promote cold modes of their equipment. However, care must be taken as these devices produce temperatures of 80°C and more if not moved very rapidly over the tissue to avoid overheating [197]. Therefore, specific labeling of such devices is necessary as hybrid gas plasmas as suggested by Jerome Canady, as otherwise, they may be confused with true-body-temperature gas plasma devices by clinical staff and may cause unintended severe burning injuries. These hybrid gas plasmas or hybrid plasma coagulators nevertheless produce ROS [198,199]. Notably, from a physics point of view, ‘cold’ refers to gas plasma operating out of thermodynamic equilibrium with T_e_ > T_i_ > T_n_ (i.e., electron temperature is greater than ion and neutral temperature), while ‘hot’ plasma refers to fully-ionized gas without neutral spcies (T_e_ = T_i_). From that angle, plasma devices with 200°C and more can be still considered ‘cold’ in physics, as long as the thermodynamic equilibrium is not met. In this review, however, the term ‘cold’ is used in the biological sense, i.e., not tissue-damaging, while ‘hot’ intuitively translates to damages or potentially damaged tissues.

### 4.1 Patient-derived cancer tissues

Tumor biopsies are a suitable tool to analyze and understand the response of cancer tissue to a given treatment modality. Biopsies fully recapitulate the TME and its composition but not its dynamics due to the missing blood and lymph flow and nerval stimuli. Several studies were performed with tumor biopsies of different origins receiving gas plasma treatment ex vivo (Table 3). All studies were performed using gas plasma jets, and all studies investigated intact tissue biopsies using the approved medical device kINPen MED. Without exception, regulated cell death (RCD), mainly apoptosis, but no necrosis or abnormal tissue morphologies were observed. Apoptosis was the highest at tissue sites exposed directly to gas plasma jets, while the opposite side remote to the gas plasma treatment was unaffected. This underlines the relatively low penetration depth of single gas plasma treatments, with acute toxicity only occurring within the direct treatment zone. However, it has been established that gas plasma-induced tissue effects can propagate further than within the range of acute effects (e.g., toxicity) being observed. For example, increased deep-tissue oxygenation has been found using hyperspectral imaging of both non-malignant skin and tumor tissue follow gas plasma treatment [200,201]. How signaling into deeper tissue regions following gas plasma exposure is realized has not been elaborated yet. Potential candidate pathways are cell-cell communications via junctional proteins or the release of mediators of inflammation, such as cytokines, chemokines, and growth factors. About the latter, ex vivo gas plasma treatment of primary tumor tissues has been found to change the secretion profiles markedly when compared to untreated controls if the tumor tissue was cultured for several hours post gas plasma exposure. This was found for all tumor types investigated, i.e., melanoma [194], cutaneous SCC [196], basal cell carcinoma (BCC) [196], glioblastoma [195], breast cancer [202], and urothelial (bladder) cancer [203]. Transcriptomic analysis of the latter also revealed that such changes are the consequence of changed signaling pathways induced by gas plasma treatment rather than merely reflecting the loss of, e.g., the number of viable cells that would participate in cytokine secretion and binding and/or uptake. Albeit more detailed immunophenotyping of such ex vivo gas plasma treatment is awaited, it can be anticipated that apoptosis is not only confined to tumor cells but also other cell types in the TME, including leukocytes (immune cells) and stromal cells. For instance, fewer FOXP3^+^ regulatory T-cells (T_reg_) were identified in gas plasma-treated patient-derived SCC tissues, presumably due to their apoptotic demise [196]. This questions the idea of selectivity of gas plasma on cancer cells, that is also not clear from the *in vitro* results described above, and raises three points. First, selectivity is critical for systemic treatments such as chemotherapy, where all body cells are exposed to and affected by the agent in question. This is less perilous for local treatment modalities, such as cryoablation, radiotherapy, electrochemotherapy, hyperthermia, and even surgery, that may affect or remove all malignant and non-malignant cell types in the TME. Nevertheless, these treatments only have limited consequences systemically, unless they show immune-stimulating features. Second, damage and inflammation are prerequisites for ICD and anticancer immunity [204], making a certain potency of gas plasma exposure necessary to potentially induce such processes. Third, most resident non-tumor cell types in the TME are specifically attracted and hijacked by cancer cells to promote growth-conserving and -promoting tasks. This is true for, e.g., cell types such as cancer-associated fibroblasts (CAFs) [205], T_reg_ [206], M2 macrophages (also called tumor-associated macrophages, TAM) [207], N2 neutrophils (also called tumor-associated neutrophils, TAN) [208], and tumor-associated endothelial cells [209]. The damage or removal of such cells by local treatment modalities such as gas plasma may be highly desired to make the treatment more durable by disallowing the cells to promote tumor cell regrowth, especially in tumor types rich in immunosuppressive and stromal cells, such as pancreatic cancer and glioblastoma.

**Table 3:** Overview of gas plasma treatment studies on patient-derived tumor tissues exposed ex vivo.

malignancy	# of pat.	gas plasma source /gas /treatment time	main finding following gas plasma treatment	ref.
basal cell carcinoma	8	kINPen MED (neoplas); argon; 120s	significantly increased apoptosis and granzyme and IL-17A release; significantly decreased regulatory T-cells (FOXP3^+^) and IL-1β, G-CSF, GM-CSF, and CCL5 release	[196]
breast cancer	10	helium plasma jet (non-commercial), 240s	significantly increased dead cells, caspase 3 activation, e-cadherin expression, and IFNα2, IL-17A, IL18, and IL33 release; significantly decreased IFNγ, IL6, IL8, and MCP1	[202]
breast cancer	20	argon plasma jet (non-commercial) 300s	significantly increased apoptosis and significantly decreased number of CD163^+^ cells	[392]
cutaneous squamous cell carcinoma	8	kINPen MED (neoplas); argon; 120s	significantly increased apoptosis; significantly decreased CCL5, PDGF-aa, and GM-CSF release	[196]
glioblastoma	16	kINPen MED (neoplas); argon; 120s	significantly increased apoptosis and decreased b-NGF, IL-6, sTREM-2, TGFβ, TNFα, and TREM-1 release	[195]
head and neck cancer	10	kINPen MED (neoplas); argon; 30s	significantly increased cytochrome c release in gas plasma-treated HNSCC but not non-malignant (healthy) tissue; non-significant differences regarding apoptosis	[62]
melanoma	6	kINPen MED (neoplas); argon; 120s	significantly increased apoptosis; significantly decreased IL-10, VEGF, and CCL2, and increased CCL5 and EGF release in ≥2 patients	[194]
melanoma	5	kINPen MED (neoplas); argon; 120s	correlation between xCT (SLC7A11) and S100 and caspase 3 activation in terms of gas plasma treatment	[393]
oral squamous cell carcinoma	20	argon plasma jet (non-commercial) 300s	significantly increased apoptosis	[392]
urothelial/bladder cancer	16	kINPen MED (neoplas); argon; 120s	significantly increased apoptosis, transcriptomics of untreated and gas plasma-treated healthy and tumor tissue revealed FGFR3 and AIFm2 as response genes	[203]

Regarding future work, it must be stated that primary cancer tissues are difficult to work with. They show a high degree of heterogeneity between patients and different tumor biopsy locations of the same tumor vary in terms of TME composition and degree of necrosis. To visualize the effects of gas plasma treatment, biopsy incubation is required to allow biological processes (transcriptional, translational, expression, and cellular changes) to take place following extended gas plasma-derived ROS exposure, which may be tedious in the laboratory depending on the type of tumor tissue and, e.g., degree of contamination (e.g., colon cancer). Notwithstanding, three major questions can be addressed using gas plasma treatment of patient-derived tumor tissue ex vivo. First, what is the penetration depth of gas plasma treatment for a given biological response extending from the tissue border extending into deeper tissues? The second question, which remains to be elucidated, is whether gas plasma treatment confers equal or differential toxicity to different cell types in the TME, i.e., does the number of killed tumor cells align with dead macrophages and T-cells, or are the latter targeted even before tumor cell inactivation sets in because of higher doses required for the tumor killing event to occur? These two points can be addressed using multicolor fluorescence microscopy of ultrathin-sectioned tumor tissues. The third point relates to the overall inflammatory environment modulated by gas plasma exposure, which can be measured conveniently in tumor tissue culture medium supernatants, giving an overview of the total secretion responses even of parts within tissues that are remote to the gas plasma exposure but may still receive secondary signals from the oxidative stress inflicted in the plasma-tissue-interphase cells. Albeit tumor tissues have been extensively profiled by omics techniques [210,211], its use in investigating gas plasma responses may be at least challenging with regard to the relatively small number of cells at the tissue border directly being affected by the treatment, the heterogeneity between biopsies, and limited possibility of such techniques and approaches to provide kinetic information, e.g., differences between early and late protein translation patterns in the same type of tissue. While there is no solution for the latter, the former issues can be addressed by increasing the number of tissues analyzed for inferring overarching rather than individual tumor tissue responses. In addition, it would be enormously helpful to be able to follow the gas plasma-derived ROS trajectories into tumor tissues in a dynamic fashion, giving unpreceded information on gas plasma devices, treatment times, feed gas adaptations, treatment distances, and many others. Yet, adequate research tools have not been developed yet in the field of redox biology and chemistry or beyond to allow for this.

### 4.2 Case reports and clinical studies

Clinical evidence of effective cold gas plasma application in cancer patients is scarce (Table 4). Only one published two-armed, randomized, prospective trial with an approved gas plasma device and one single-arm recruiting trial (NCT02759900) with an experimental gas plasma device are available, while the other studies are case reports or series. So far, the two cancer patient cohorts subjected to gas plasma treatment were those suffering from topical cancer lesions. One is actinic keratosis (AK), a carcinoma in situ, which can lead to more deleterious invasive squamous cell carcinoma (SCC) [212]. The other is ulcerating oral and oropharyngeal SCC, also called head and neck cancer (HNSCC) [213]. There are many therapeutic options against AK, each with its benefits and pitfalls [214].

**Table 4:** Overview of case reports and clinical trials in gas plasma cancer patient therapy.

malignancy	# of pat.	plasma source/gas	trial #	trial status	main findings following gas plasma treatment	ref.
actinic keratosis	60	SteriPlas (Adtec); argon; 1-24x 180s	–	prospective, randomized trial	intrapatient comparison of gas plasma vs. diclofenac; significantly fewer lesions (week 12) lesional area with gas plasma; no adverse events	[218]
actinic keratosis and others	tba	floating-electrode DBD (non-commercial); not stated	NCT02759900	recruiting	up to 100 patients with actinic keratosis, acne/rosacea, verruca plana, warts, tinea, Bowen’s disease, etc. to receive gas plasma exposure	–
actinic keratosis	12	SteriPlas (Adtec); argon; 1-12x 120s	–	case series	Complete remission in 2 patients; significantly decreased number of lesions; lesion size, and AKASI scores; no adverse events	[217]
actinic keratosis	1	Maximum electrosurgery beamer (KLS Martin); argon; 1x 60s	–	case report	inflammation followed by full regression and healing of the lesional wounds; no adverse events	[216]
actinic keratosis	4	SteriPlas (Adtec); argon; 7x 120s	–	case series	total number of lesions decreased in 6 areas; promising responses with clinical downgrading in the Olsen scale; no adverse events	[226]
actinic keratosis	5	floating-electrode DBD (non-commercial); 1x 60-120s	–	case series	17 lesions in 5 patients; 9 lesions showed full clinical resolution, 3 improved, 5 no effect; no adverse events	[215]
head and neck cancer	6	kINPen MED (neoplas); argon; 30-60s /cm^2^	–	case series	6 patients with advanced oropharynx SCC received gas plasma palliative therapy successfully	[219]
head and neck cancer	21	kINPen MED (neoplas); argon; 30-60s /cm^2^	–	case series	microbial and partially tumor reduction in palliative HNSCC patient gas plasma therapy of	[220]
head and neck cancer	2	kINPen MED (neoplas); argon; 30-60s /cm^2^	–	case series	hyperspectral imaging in palliative HNSCC patient gas plasma therapy of showing increased superficial and deeper cutaneous oxygen saturation and hemoglobin concentration	[200]
head and neck cancer	12	kINPen MED (neoplas); argon; 30-60s /cm^2^	–	case series	palliative HNSCC patient gas plasma therapy led to partial remission in some patients and decreased request for pain medication and typical odor	[394]
head and neck cancer	10	kINPen MED (neoplas); argon; 30-60s /cm^2^	–	case series	palliative HNSCC patient gas plasma therapy came with no severe side effects	[228]

Gas plasma therapy worked very well and was well tolerated with no side effects in AK patients [[215], [216], [217]]; it even outperformed diclofenac standard treatment in a head-to-head trial [218] and could be a cost-effective addition to the clinical AK treatment repertoire. For HNSCC, a series of end-stage patients have received gas plasma as palliative therapy [200,219,220]. The primary objective was to decrease the microbial burden of these ulcerating tumors, as serious infections interfere with effective palliation measures. The antimicrobial effects of the gas plasma treatment were successful. Surprisingly, some patients had a significant tumor mass decrease after several gas plasma treatment sessions, which continued over several months of frequent gas plasma therapy before tumor growth finally progressed. It was speculated that these responses could be linked to antitumor immune responses, as the gas plasma-killed microorganisms may have served as an adjuvant with a local cancer cell inactivation and freeing of tumor antigens [221]. Thus, gas plasma therapy may aid in palliating patients suffering from ulcerating tumors. Yet, the tool seems unsuitable for large tumor mass debulking, and there would also be few indications to do so. As suggested by one *in vivo* study [164], another potential application is intra-operative tumor wound gas plasma care as suggested, i.e., the gas plasma treatment of surgical margins after tumor removal to reduce micro-metastases and decrease the risk of cancer recurrence. There is one recruiting clinical trial (NCT04267575) with this aim but, unfortunately, this trial is single armed (i.e., not intended to compare tumor wound margins with and without gas plasma treatment) and there is no interim analysis published so far. Besides gas plasma treatment of established tumors, there is the idea of treating precancerous conditions that, if left untreated, may lead to invasive tumor growth. This applies to, for instance, oral inflammatory or potentially malignant disorders such as leukoplakia and oral lichen planus (lichen ruber). For the latter, one study is available describing the inflammatory profile and T-cell viability in ex vivo gas plasma-treated lichen ruber samples compared to healthy control oral mucosa tissue, along with a case report of a patient highly benefiting from intra-oral gas plasma therapy [222]. For leukoplakia, also promising results have been achieved [223]. Another premalignant disorder is cervical intraepithelial dysplasia (CIN). This sometimes inflamed, abnormal tissue is often associated with vaginal Human Papilloma Virus (HPV) infections [224]. Up to 65% of cases heal spontaneously, while about 10% progress from the low-grade form CIN2 to the high-grade form CIN3 [225]. In a single-arm interventional clinical trial (NCT03218436), an electrosurgical argon plasma device was used for the superficial lesion treatment of 20 patients during colposcopy [198]. Although the hybrid gas plasma used was relatively hot with tissue heating up to 80°C, tissue burning can be avoided if the device is moved fast enough over the tissue surface. Full, durable CIN remissions were 90% after gas plasma treatment compared to up 65% spontaneous remission, making this gas plasma application potentially interesting for a presumably milder treatment of CIN compared to more painful standard cryo-ablation techniques. Notwithstanding, two-thirds of patients reported mild-to-modest side effects with argon plasma electrosurgical treatment used in the ‘cold’ plasma mode as well. Main conclusions are summarized in Box 2.

Box 2 **Main conclusions from gas plasma cancer treatment in vitro studies.**

⁃ The different types of gas plasma sources exert anticancer effects via similar mechanisms and dissimilar scales (exposure times; energies)
⁃ The predominant cancer cell death modality is apoptosis
⁃ Excess liquid serves as ‘radical sink’ where the many different short-lived gas plasma-derived reactive species form a few long-lived species (H_2_O_2_ and NO_2_^−^/NO_3_^−^, or OHCl)
⁃ Gas plasma-tumor cell inactivation with most devices majorly depends on H_2_O_2_, as shown by catalase abrogating many effects *in vitro*
⁃ The sensitivity of tumor cells to gas plasma-induced inactivation varies up to 2 orders of magnitude
⁃ Gas plasma-induced oxidative cell death is not tumor cell-selective but depends on the cell types' capability of coping with oxidative stress
⁃ Gas plasma can elicit hallmarks of immunogenic cell death (ICD) to promote immune-recognition of cancer cells

Alt-text: Box 2

## 5 Quo vadis, experimental and clinical gas plasma cancer treatment

### 5.1 Gas plasma devices for oncology

When taking into consideration gas plasma devices approved for medical, not cosmetic, conditions and being genuinely non-thermal (cold), there are only a few devices on the market that are off-label testable for anticancer applications and were reported for such applications, mainly actinic keratosis and palliative HNSCC patients [18,200,[215], [216], [217], [218], [219], [220],[226], [227], [228]]. There is a need for larger and randomized trials to raise evidence on the beneficial role of gas plasma treatment in such diseases and to complement the existing therapy portfolios. As an outcome, cancer treatments could be amended as another medical indication for which the gas plasma devices are approved for. This would propel the field of gas plasma oncology in general, and the gas plasma device that had received this approval in particular, and would spur new clinical interest in using the technology for several types of tumors and novel investigator-driven clinical trials. Alternatively, a well-documented and accepted off-label use of gas plasma in medicine might also be conceivable, especially since there is more than a decade of medical experience with gas plasma technology in medicine, particularly dermatology. To date, frequent severe adverse events or long-term damage in patients due to gas plasma therapy has not been reported, neither in the literature nor anecdotally. Mainly in the first scenario, but also in the second scenario (in the case of the European Union), CE-marked and/or approved gas plasma devices would benefit immediately from increasing clinical experience. The gas plasma devices mainly used for basic research, which is the vast majority of devices described in the literature, may – despite any promising evidence – likely not be getting approval because these procedures, at least in the EU, requires extensive documentation on safety and efficacy according to the medical device regulation (MDR). In short, only those devices can make a clinical impact that are approved, and the fewer devices are approved, the more clinical experience will be gained with the few approved devices available. Even dramatically anticancer effective basic research gas plasma devices will not transition to medicine without approval processes. At the same time, it is unclear to what extent gas plasma sources would differ in their effectiveness in medical applications as there are no clinical head-to-head comparison studies available.

Vice versa, from a basic science perspective, the polypragmasia found in using different gas plasma devices, geometries, feed gas types, excitation frequencies, treatment times and energies, and ROS-generation profiles generate a fruitful pool of new approaches to potentially complement existing clinical strategies. For gas plasma-treated liquids, the bottleneck is that the unique short-lived gas plasma chemistry delivered to the tumor target is not adequately mimicked by liquid-dominated cell culture systems in which long-lived oxidants are quickly formed from short-lived ROS. Those long-lived oxidants not only are easily added by chemical means at a lower economic burden, but they also do not recapitulate the short-lived dominated gas plasma reaction chemistry at tumor tissue sites, albeit this argument is extrapolated from biomolecule studies [132,[229], [230], [231], [232], [233], [234], [235]]. Hence, the clinical relevance andn significance of gas plasma-treated liquids is uncertain as of now. For exploring novel direct gas plasma applications in oncology, tumor tissues should be included in the research strategy. Such tissues could be three-dimensional tumor spheroids [[236], [237], [238], [239]], *in ovo* xenograft organoids [142,169,203,240,241], patient-derived organoids [[242], [243], [244]], patient-derived tumor tissue (Table 3), and/or animal models (Table 2). Another model that allows the analysis of spatial (3D) biology is hydrogels, especially when embedded with tumor cells, to follow the gas plasma trajectory of cell death within the hydrogel by using confocal laser scanning fluorescence microscopy and spatial image quantification approaches [43]. In addition, it may be useful to compare the biological responses of a new gas plasma device by benchmarking it within the same assay system against an established gas plasma device. In biological gas plasma sciences, such approaches are scarce as each laboratory and group usually investigates its own historical devices. However, a recent, very thorough comparison of redox chemistry, tumor toxicity, immunogenicity, immune-cell activation, and genotoxicity between a novel dual-jet against the kINPen provides hope that such approaches may be utilized more intensively in the future [169].

### 5.2 Gas plasma research models in oncology

**Fig. 5:**
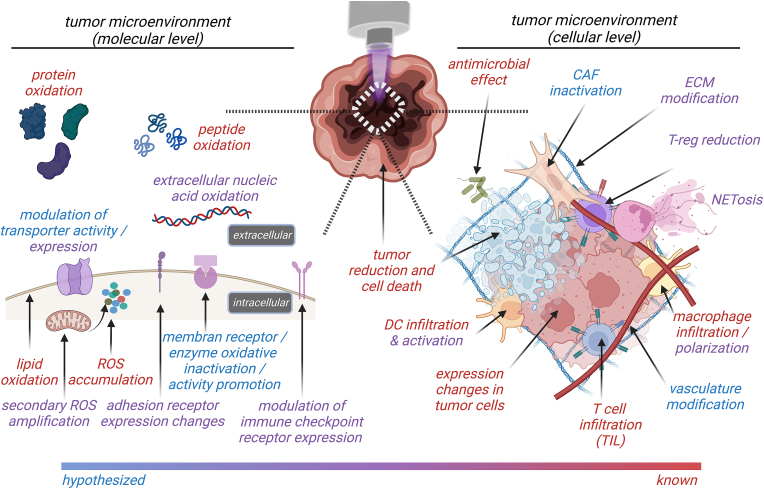
Mechanisms and mode of action in gas plasma-treated cancers and cancer cells. Colors indicate evidence level (see color-scale bar at the bottom).

Congruent to other areas of cancer research, several levels of detail are investigated in gas plasma cancer treatment (Fig. 5). Albeit being the most difficult to obtain and to work with, proof-of-concept studies with ex vivo investigated primary patient-derived tumor tissue are the first convincing milestone of gas plasma therapy to be potentially effective in the tested type of tumor with the maximum TME complexity available. Simultaneously, it infers about potential gas plasma penetration depths and tissue abnormalities possibly not visible in the mouse model due to, e.g., extensive fur or differences in skin physiology. Information on the role of, e.g., stromal cells and penetration depth can also be obtained in the i*n ovo* model [245], which is ideally suited for gas plasma cancer studies [246]. This is heavily needed, as the lack of knowledge on bystander, immunosuppressive, and stromal cells in gas plasma-treated tumor tissue has been identified [247], but the number of studies specifically performing *in ovo* co-cultures or using syngeneic mouse models followed by targeted and detailed intra-tumoral cell type investigations is scarce. At this cellular level within the TME, the most gain of knowledge can be expected in the field of gas plasma cancer science because those types of studies also propelled cancer immunotherapies from basic science to clinical translation in the past decade [[248], [249], [250]]. What would be especially fascinating to investigate is the amplitude of impact of gas plasma-inactivated tumor-supporting cells, such as CAFs, TAMs, TANs, and T_regs_. Laboratory three-dimensional models may allow setting up such tumor cell-stromal cell co-cultures, as demonstrated elegantly recently with stellate cells [241] and dendritic cells [142], to assess their differential effect on the tumor cells and tumor growth following gas plasma exposure. Such cells are likely affected directly by gas plasma exposure, but apart from descriptive findings, the details on effects and mechanisms remain to be elucidated. Last but not least, gas plasma-derived ROS likely change the function and appearance of the extracellular matrix (ECM) in tumors. Sophisticated model systems and analysis techniques will be needed to shed light on whether and how those gas plasma-driven modifications effectively change the TME cells’ behavior, including biochemical studies on the ECM’s composition and its (ox)PTMs.

From such subcellular, molecular, and/or biochemical perspectives, several aspects are critical to understanding the functional consequences of gas plasma cancer treatment. For instance, immune-checkpoint expression patterns are known to associate with therapy outcomes in cancer patients [[251], [252], [253], [254]], and while a few studies have addressed their expression in tumor cells following gas plasma exposure [[255], [256], [257]], the *in vivo* relevance of such ROS-modified receptor and ligand pattern expression on tumor cells remains elusive. It is undoubted, however, that ROS accumulate immediately after gas plasma treatment of tumors, as recently shown for the first time in melanoma and SSC xenografts in mice [258]. It would be interesting to understand whether such ROS accumulation is similar for different tumor types exposed to the same gas plasma device or similar tumor types exposed to different gas plasma devices, and if there is a linear relationship between gas plasma exposure time and signal amplification. In general, it should be noted that gas plasma technology may not only be a therapeutic tool in oncology but can also be used as a multi-ROS technology to study tumor cells’ behavior under short-lived ROS-induced oxidative stress conditions that are more complex and may therefore resemble the *in vivo* situation much better regarding several biomolecules, including lipids. Apart from computer modeling studies [[259], [260], [261]] and experimental evidence outside the cancer context [[262], [263], [264], [265], [266], [267]], the exact role of gas plasma-mediated lipid oxidation and general effects on cell membranes in tumor control is understudied. There is first evidence of a modulation of drug and metabolism-associated membrane transporters following gas plasma exposure [72,268,269], but, again, the functional consequences for gas plasma tumor therapy are unknown. Along similar lines, extracellular biomolecules such as nucleic acids [[270], [271], [272], [273], [274]], peptides [235], and proteins or enzymes [132,264,[275], [276], [277], [278], [279], [280]] become readily modified or even inactivated following extensive gas plasma exposure. Using relatively non-complex biomolecules, such as cysteine, there is sufficient evidence of gas plasma-induced oxPTMs [230,231,234,235,262,264,281], not only showing unique patterns (Table 5) not reproducible with experimentally added long-lived oxidants, such as H_2_O_2_ or PTL [229,282,283], but also pointing to distinct functional consequences of oxPTM biomolecules potentially relevant for disease therapy or diagnostics [132,284]. Two striking examples show why the gas plasma treatment might be particularly promising. One study found that exposure of tumor cells *in vitro* with such oxidized proteins reduced their viability compared to supplementation with the native counterpart [277]. While the real-world consequences of those findings remain elusive, such an approach may circumvent the apparent limitation of gas plasma-treated liquids either being not approved as medical products (e.g., cell culture media) or its effect being dominated by H_2_O_2_. Instead, upon identifying a suitable protein candidate, the protein may absorb the chemical energy provided by the gas plasma-derived short-lived ROS by accumulating tumor-toxic modifications. Provided the ideal protein properties and toxic modifications would be elucidated and mapped (e.g., via mass spectrometry), a future approach could be injecting hyperoxidized protein directly into tumor types previously identified to react sensitively to this approach. The second study in this realm used a hen’s egg protein as a model to infer how gas plasma-induced oxPTMs change the immunogenicity of that protein [132]. Notably, a marked elevation in T-cell activity was identified, together with increased tumor control in mice when using cancer cells expressing this experimental antigen. This may open entirely new research lines for therapeutic or preventive anticancer vaccination schemes, provided that the target tumor antigen is known. The implications of this approach are vast, and many aspects remain to be discovered to exploit the full potential of this idea, as previously outlined [285].

**Table 5:** Major post-translational modifications observed on aminoacids and proteins following gas plasma exposure. Reproduced from Ref. [235].

monoisotopic mass shift [Da]	elemental composition	chemical modification (potential product)
+15.99	+O	hydroxylation (oxidation)
+31.98	+2O	dihydroxylation or peroxide (dioxidation)
+47.98	+3O	hydroxylation + peroxide, trihydroxylation, sulfonic acid (trioxidation)
+28.99	+N + O-H	nitrosylation
+44.98	+N + 2O-H	nitration
+60.98	+N + 3O-H	nitration + oxidation
+76.97	+N + 4O-H	nitration + dioxidation
+0.98	-N-H + O	deamidation
- 0.98	+N + H-O	amidation
+13.98	+O-2H	carbonylation (oxo group)
+29.97	+2O-2H	oxo group + hydroxylation
+45.97	+3O-2H	oxo groups + two hydroxylations or peroxide
- 2.02	-2H	didehydrogenation (double bond)
- 4.03	-4H	two didehydrogenation (two double bonds)
+4.98	+2O-N-C-H	ring cleavage (histidine: formylasparagine)
- 3.05	+O-5H-N	oxidative deamination (lysine) [395]
+33.96	+Cl-H	chlorination

### 5.3 Challenges and opportunities in clinical gas plasma cancer treatment

**Fig. 6:**
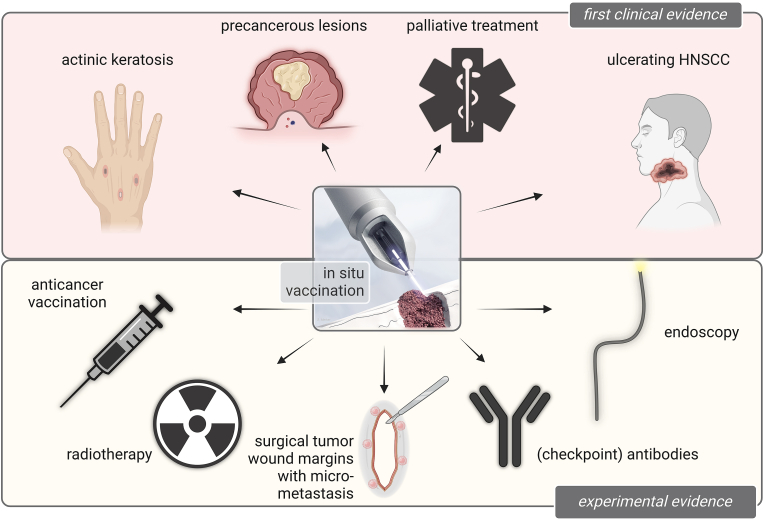
Experimentally and clinically tested gas plasma anticancer treatment modalities.

Apart from what is and has already been done clinically in the realm of gas plasma cancer treatment (Fig. 6), there are gas plasma approaches with higher and lower application potential, each with its challenges and opportunities.

Gas plasma-treated liquids may seem elegant as they can be produced in large quantities, stored, and injected into all types of tumors and body cavities. Conceptionally, however, there is a lack of evidence that medical product liquids (e.g., Ringer’s lactate, 0.9% sodium chloride) contain agents unique to the gas discharge exposure that cannot be replicated by the simple (and cheaper) chemical addition of the long-lived oxidants H_2_O_2_ and NO_3_^-^ or NO_2_^-^. To this end, their application for treating, e.g., peritoneal carcinomatosis and other malignancies might be limited by economic or regulatory (e.g., quality control) reasons [162]. Notwithstanding, such liquids would be of interest if a *battery* compound would be identified that stores the unique short-lived radical chemical energy of the gas plasma process in the form of modifications that would be unleashed upon the tumor at, e.g., a specific temperature or upon enzymatic activation. Such oxidative prodrug approaches could be interesting to several medical fields, but apart from initial studies [286], the identification of suitable candidates, treatment conditions, and gas plasma sources remain awaited.

Regarding cancer treatment *per se*, the idea of gas plasmas utilized for debulking tumor mass seems a historic misperception. In most circumstances, the surgical knife is the method of choice to remove large tumors, and what cannot be reached by the knife will likely not be in reach for gas plasma. There are exceptions where surgical removal is not meaningful, e.g., if too many metastases have been spread, and those applications are also not to be solved with gas plasma therapy. In some situations, the main tumor is too close to vital structures to be surgically removed, e.g., the *arteria carotis*, and if the tumor is on the surface, gas plasma therapy could be an option [18]. For tumors inside the body, however, where surgery is needed to reach the tumor, a single gas plasma exposure likely will not debulk significant tumor mass within a single exposure attempt, as understood based on a decade of knowledge in gas plasma cancer treatment in dozen of animal models and using different gas plasma devices. In those circumstances, established medical techniques such as thermo- and cryoablation are much more suitable [287,288]. As ROS-generating technique, radiotherapy can be used, which can be applied with high precision, targets deep tissues, and can be applied repeatedly (fractionation) [289,290]. In addition, studies provided evidence of a good combinatorial potential of radiotherapy with gas plasma therapy [255,291]. Hence, albeit not as a curative attempt, gas plasma therapy may be useful for the palliation of patients with large, ulcerative, therapy-resistant tumor sites, e.g., skin cancer (cutaneous SCC, melanoma), breast cancer, and oral SCC. In these situations, frequently repeated gas plasma exposure is amendable to control, for instance, tumor infections and/or growth to support palliation, and potentially also provide immuno-stimulation. In the clinic, however, head and neck cancer cell adaptation to gas plasma therapy-induced tumor toxicity was also observed [221], and frequent gas plasma-induced oxidative stress can select for slow-cycling tumor cells with improved stress resistance [292]. Such adaptation and reduced therapy responses, however, has been observed for most cancer therapies utilized in the clinic, and are not specific to gas plasma therapy *per se*.

Propelled by the Nobel Prize in Medicine and Physiology in 2018, the topic that has received the by far the most attention in the last 5 years of oncology is cancer immunotherapy [293]. In the narrow sense, cancer immunotherapy refers to the injection of antibodies or (specialized) immune cells in the patient to promote existing or even elicit new antitumor immune responses on the systemic level. According to the U.S. National Cancer Institute, “Immunotherapy is a type of biological therapy. Biological therapy is a type of treatment that uses substances made from living organisms to treat cancer.” Hence, a local therapy such as radiotherapy (or gas plasma treatment) is not a cancer immunotherapy *per se*. However, it can be proposed that therapeutic technologies such as gas plasma can support cancer immunotherapeutic schemes. There are three major possibilities for achieving this (Fig. 6).

The first involves the post-surgical gas plasma treatment of the tumor wound resection margins. This idea has been around for over a decade, but experimental evidence was scarce, except for a recent *in vivo* study [164]. This approach could be positive two-fold, by reducing micro-metastases in the remaining tissue to reduce tumor relapse and potentially induce ICD and by that induce priming of the patients’ antitumor immunity. With several gas plasma devices already being approved for wound decontamination, routine post-surgical gas plasma care could be easily argued to prevent wound infection today based on approved indications using the certified devices [6]. Thus, the bottleneck is finding determined tumor surgeons willing to apply this regularly and document the results, i.e., the number of relapses at that site. This could be linked to the prediction from the pathologists’ identifying the resection being either R1 or R0. Since gas plasma has a very good risk profile, it can be anticipated that even if only a minor fraction of patients would benefit from the treatment, the benefits would outweigh the risks. Another approach is related to this, i.e., gas plasma therapy of ulcerating tumors, which are often infected, as outlined above. Here, the infection may serve unwillingly as adjuvants to prime anticancer immunity against tumor antigen provided by the gas plasma exposure in a therapeutic in situ vaccination scheme, as hypothesized with the case series of patients suffering from ulcerating, infected head and neck cancers [221]. Closely related to this, endoscopic gas plasma devices may be used to stimulate such locally confined immunogenic tumor cell damage at all body sites reachable via endoscopes. Gas plasma technologies small enough to be employed within endoscopic channels were developed years ago by Eric Robert and colleagues at GREMI (France) and recently complemented at INP (Germany) [294,295], and are waiting to be employed in cancer research apart from *in vitro* toxicity studies [296].

The second approach involves autologous anticancer vaccination. Two promising *in vivo* reports [37,136] fetched the idea of using gas plasma-treated cancer cells as a vaccine, showing that subsequent tumor growth upon injection of live tumor cells was reduced due to promoted antitumor immunity. The same is possible with specific gas plasma-treated proteins [132]. Hence, one vision could be to use surgically removed or biopsied tumor tissue, utilize it within a gas plasma process, and inject it back into patients as an autologous antitumor vaccine. *In vivo* studies are awaited on preventive and therapeutic vaccination trials to provide proof-of-concept data that gas plasma-treated autologous anticancer vaccination schemes provide therapeutic benefits, as outlined previously for its potential and risks [285]. This would be combined with the third approach of combining either direct gas plasma treatment of post-surgical tumor margins or ulcerating tumors with a later injection of immune checkpoint inhibitors. This would help support antitumor T-cells being formed later after the gas plasma treatment. Similar treatment schemes have also already been proposed and partially employed following radiotherapy [297], and a analogous rationale can be hypothesized for gas plasma cancer treatment and immunotherapy. Regarding radiotherapy, it would be interesting to understand the combination potential of both technologies regarding topical tumors, as there could be a synergistic liaison between intracellular and extracellular cancer ROS propagation for therapy and immuno-stimulation.

**Fig. 7:**
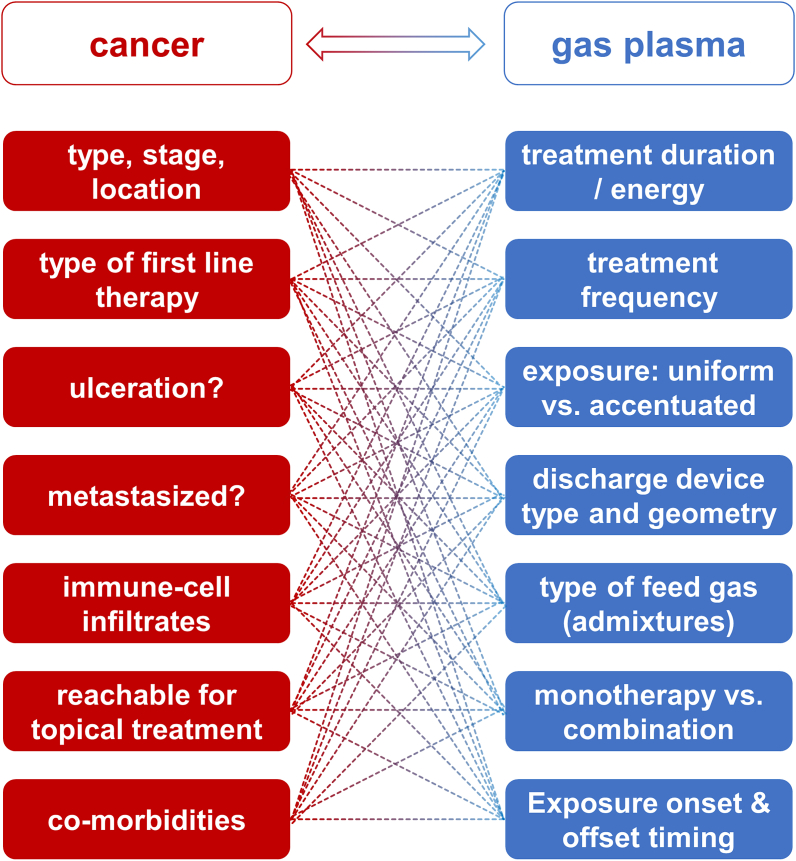
Experimentally and clinically tested gas plasma anticancer treatment modalities.

In general, a plethora of gas plasma combination approaches with chemotherapies, targeted therapies, and immunotherapies is conceivable for oncology. The quest will be to identify the most promising approaches and, ideally, the best performance parameters within an extensive iteration matrix (Fig. 7). In oncology, there are consensus-based first-line therapies based on, for instance, tumor entity, stage, and molecular subtype (e.g., breast cancer [298]). As outlined above, it is also decisive where the tumor is located and whether it is ulcerating, apart from patient-individual parameters such as immune fitness and co-morbidities that are known to affect therapeutic outcomes of approved treatments, such as immune checkpoint inhibitors. With regard to the gas plasma device, many parameters may be more or less important for clinical anticancer treatment; the actual relevance of each of the parameters to contribute to clinical responses is unknown. It appears reasonable that longer treatment times may exert greater effects, but it is unclear, for instance, whether tumors can become overdosed with gas plasma and how this would affect the patient (and the tumor). A dose escalation study on clinical gas plasma cancer treatment has not been published. Along similar lines, it is unknown how often gas plasma treatment should take place – in rodent cancer models, usually 1–4 times a week was reported [37,132,149,164,183,258,[299], [300], [301], [302], [303], [304]]. A high gas plasma treatment frequency appears plausible because gas plasma-derived ROS do not persist as drugs would, the gas plasma treatment is mild and well-tolerated, and the direct penetration depth of the treatment is limited, implying multiple treatment sessions being needed to spur greater anticancer effects. Simultaneously, it is unknown when gas plasma therapy should be started or stopped within clinical routines. Related to this, the question is whether the gas plasma exposure should be done uniformly across the entire tumor surface or if there would be a rationale for exposing some dedicated regions for longer treatment times in an accentuated fashion. For instance, deliberately necrotic areas might benefit less from gas plasma therapy due to extensive cell death and tissue destruction being present in such areas already. Gas plasma monotherapy for cancer treatment might be useful in patient palliation or widespread actinic keratosis, while it would be interesting to see if gas plasma combination treatments may potentiate existing therapies, especially within in situ or autologous vaccine approaches (Fig. 6). Concerning the type of gas plasma source and its geometry as well as its feed gas or potential feed gas admixtures, it cannot be stated whether some devices may be optimal for a given set of tumors while others may be optimal for another set of tumor entities. From the *in vitro* results and general findings of reactive species analysis, all gas plasma devices share similarities regarding effects and effectors. At the same time, it is clear that not all devices are equally effective, mainly because there has been no agreement in the community on a unifying concept of a gas plasma dose to calibrate devices and efficacies against each other and thus make them comparable. However, this academic debate is secondary to the fact that clinical evidence is scarce that gas plasma plays a role in generating real patient benefit, while preclinical evidence is solid. Therefore, and disregardless of the many parameters and uncertainties (Fig. 7), it is critical to start gaining clinical evidence in oncological situations that may require an additional tool to support tumor control and patient health. At least from the perspective of the safety of gas plasma applications, there is a consensus on a lack of severe side and genotoxicity from over ten years of regular clinical experience with the 3–4 approved gas plasma devices in central Europe. Main conclusions are summarized in Box 3.

Box 3 **Challenges and opportunities of gas plasma in clinical oncology.**

⁃ Experimental use of gas plasma technology in oncology was successful for actinic keratosis patients and in the palliation of head and neck cancer patients
⁃ Apoptosis is the main mode of tumor-cell inactivation within cancer tissue, as shown by malignant lesions exposed to gas plasma in patients or ex vivo
⁃ An antitumor-selectivity, a theme with lower relevance in local treatment modalities based on physiological mechanisms, has not been shown in gas plasma-treated tumor tissues
⁃ Gas plasma-mediated inactivation of stromal and immunosuppressive bystander cells may be beneficial but needs more experimental evidence
⁃ The utilization of gas plasma-treated liquids in oncology is questionable, as substitution with chemical long-lived oxidants yields similar effects, lower costs, and higher practicability
⁃ The first overarching need is more studies on gas plasma combination treatments with first-and second-line treatment modalities specifically utilized for each tumor entity
⁃ The second overarching need is more studies on empowering anticancer immunity using gas plasma technology within in situ and therapeutic vaccination schemes and by employing syngeneic animal models and immunophenotyping approaches
⁃ The greatest bottleneck in gas plasma cancer treatment is the low number of clinical studies available

Alt-text: Box 3

## 6 Conclusions

Clinical gas plasma treatment of cancer patients is still in its infancy. The technology is exceptionally well tolerated by patients due to its body-temperature nature. Meanwhile, with ROS being the primary biological gas plasma agent, the effective anticancer effects often require repeated treatment sessions, making topical tumors such as skin and ulcerating head and neck or breast cancers logical targets, in which promising clinical results were already achieved following gas plasma exposure, especially in cancer patient palliation and treatment of actinic keratosis. Two promising applications with auspicious preclinical data are intra-operative cancer wound margin gas plasma treatment to decrease tumor recurrence and the promotion of anticancer immunity by using gas plasma-modified anticancer vaccines and in situ gas plasma vaccination potentially combined with cancer immunotherapy, such as checkpoint antibodies. Here, gas plasma technology may aid in providing additional freeing of tumor antigens in a sufficiently inflammatory and pro-immunogenic context. Further development of the technology, e.g., within endoscopy-supported anticancer treatment, may support the identification of specific niches in which gas plasma may usefully complement existing therapeutic schemes in oncology. To identify the roles of specific sets of reactive species for anticancer and immuno-promoting effects, a better characterization of reactive species profiles and their variation for identifying their specific effects is needed. In addition, there is an urgent desire to develop sensors and tools that can unambiguously identify individual types of reactive species from ROS mixtures, such as generated via gas plasma technology.

## Funding

The research of Sander Bekeschus’ group is or was funded by the German Federal Ministry of Education and Research (BMBF; grant numbers 03Z22DN11, 03Z22Di1, 03Z22D511, 03COV06A, and 01KI2125A), the 10.13039/501100001659German Research Foundation (DFG, grant number BE5801-7-1), the German Head and Neck Cancer Foundation, the Ferdinand-Eisenberger Foundation (Germany, grant number GeN1FE-20), the Gerhard-Domagk-Foundation (Greifswald, Germany), European Social Fund (ESF) in conjunction with the Federal State of Mecklenburg-Vorpommern (Germany, grant number 14-BM-A55-0006-18), the 10.13039/100014803European Food Safety Authority in conjunction with the 10.13039/501100000921European Cooperation in Science and Technology (COST Action PlasTHER, grant number CA20114), and the Marie Skłodowska-Curie Actions on Doctoral Network (MCSA-DN) *PlasmACT*.

## Declaration of competing interest

Sander Bekeschus has no conflict of interest to declare.

## Data Availability

No data was used for the research described in the article.
